# Water Films: The Motor of Phase Transitions in Salt Mixtures

**DOI:** 10.1021/acs.langmuir.5c02607

**Published:** 2025-11-13

**Authors:** Shaoheng Wang, Sebastiaan Godts, Amelie Stahlbuhk, Michael Steiger

**Affiliations:** † Department of Chemistry, 14915 University of Hamburg, Hamburg 20146, Germany; ‡ 194936 Royal Institute for Culture Heritage (KIK-IRRA), Brussels 1000, Belgium

## Abstract

The interaction of water vapor with soluble electrolytes has been comprehensively studied due to their ubiquity in nature and industry. Enormous experimental works have elucidated the mechanisms of water molecule adsorption, layer growth, and ion solvation in the water film on a NaCl crystal surface. In this study, we employ a new method to investigate the moisture uptake behavior on the NaCl crystal surface under varying humidity levels by using environmental scanning electron microscopy coupled with energy-dispersive X-ray spectroscopy (ESEM-EDX). Additionally, water vapor sorption, ESEM, and Raman microscopy are used to examine the role of water films in facilitating phase transitions and chemical reactions of salt mixtures. Our results indicate that the water uptake on a NaCl crystal surface initiates early and increases with rising humidity. A sudden increase in the condensation rate above 60% RH suggests ion solvation in the water film. In ternary mixtures, the water film occurs by water vapor adsorption or capillary condensation at the interface between two crystals in an early stage, where ions from both sides dissolve into the interfacial water film to form a nanoscale brine film. This process initiates the mutual deliquescence of a NaCl–KCl mixture at a relative humidity lower than the deliquescence humidity of either of the two single salts. For the solid–solid reaction NaNO_3_ + KCl → NaCl + KNO_3_, the process involves the dissolution of reactants and the precipitation of products, where the interfacial water film acts as a liquid bridge, promoting the ion diffusion and exchange between the two reactants.

## Graphical abstract

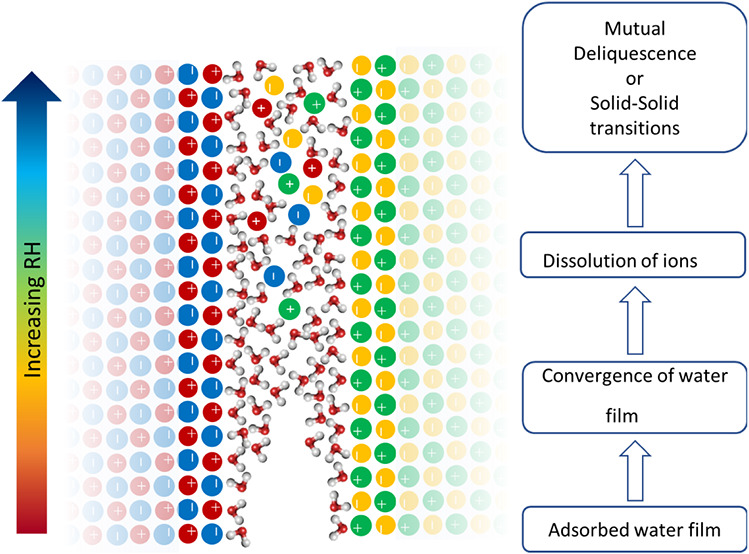

## Introduction

Water vapor sorption on a crystal surface is a common phenomenon in natural environments significantly influencing the physical and chemical stability of salts and their mixtures and is, thus, relevant in various fields of research and application, including atmospheric aerosols,^1,2^ the Martian water cycle,^3,4^ food storage,^5^ and powder engineering.^6,7^ Being the most prevalent salt in nature, the water adsorption on NaCl surfaces upon increasing humidity has been extensively studied using techniques such as infrared spectroscopy (IR),^8–10^ X-ray photoelectron spectroscopy (XPS),^11,12^ and atomic force microscopy (AFM).^13–15^ Initially, at low relative humidity (RH), a nanoscale water film on a crystal surface consists of adsorbed water molecules, which gradually evolves into a brine film with dissolved ions with increasing RH, a process known as predeliquescence.^10^ The presence of this liquid film on the crystal surface not only induces the deliquescence of soluble electrolytes and causes powder agglomeration,^6^ it also facilitates solid–solid reactions of salt particles, such as hydration reactions.^16,17^ This process involves the dissolution of the educt phase and the nucleation of the product phase within the nano- or microscale water film.

Numerous studies have investigated nanoscale interfacial reactions in various systems using advanced techniques. For example, Peng et al.^18^ and Chen et al.^19^ reported the dissolution of NaCl in ice layers below −100 °C using scanning tunneling microscopy (STM). Additionally, Putnis and Putnis proposed an ion exchange mechanism involving coupled dissolution–precipitation in the nanoscale interfacial region of a mineral and an aqueous fluid.^20–22^ These studies have primarily focused on microscale salt–water interfacial interactions in binary salt-water systems. However, in multicomponent systems, exposure to water vapor often leads to more complex phase transitions and chemical reactions, such as the mutual deliquescence of salt mixtures, the formation of double salts or solid solutions, and other water film-facilitated chemical reactions. The underlying mechanisms driving these processes are insufficiently understood. Although capillary condensation has been proposed to explain the mutual deliquescence of salt mixtures in contact with water vapor,^5,23,24^ direct experimental evidence on the specific role of a water film in this process is still lacking. Therefore, it is essential to experimentally elucidate the initiation and progression of these reactions in salt mixtures upon exposure to water vapor.

In this study, dynamic water vapor sorption, Raman spectroscopy, and environmental scanning electron microscopy (ESEM) were used to probe the role of the water film in phase transitions of a reciprocal quaternary salt system, Na^+^–Cl^–^–K^+^–NO_3_^–^–H_2_O. Our goal was to elucidate the contribution of the water film to the mutual deliquescence of the ternary salt mixtures, and, for the first time, to understand its role in the transformation of the metastable salt pair according to KCl + NaNO_3_ → NaCl + KNO_3_. The influence of relative humidity of the latter solid-state reaction is particularly interesting, as water is not involved in the reaction equation. The findings provide a detailed explanation of the mechanisms of vapor–solid and solid–solid reactions in analogous systems.

## Experimental Section

### Materials

Commercially available sodium chloride (NaCl, >99%, Roth, Germany), potassium chloride (KCl, >99.5%, Merck, Germany), sodium nitrate (NaNO_3_, >99%, Roth, Germany), and potassium nitrate (KNO_3_, >99%, Roth, Germany) were used without further purification. Additionally, single crystals of NaCl and KCl were prepared by slow evaporation of nearly saturated solutions and were subsequently used in microscopic observations.

### Water Vapor Sorption

Water vapor sorption isotherms of ten samples (NaCl, KCl, NaNO_3_, KNO_3_, NaCl + KCl, NaCl + NaNO_3_, KNO_3_ + KCl, KNO_3_ + NaNO_3_, KCl + NaNO_3_, and NaCl + KNO_3_) were measured using an SPSx-1 μ moisture sorption analyzer (ProUmid GmbH). In the SPSx-1 μ up to 23 samples are placed in dishes in a temperature and relative humidity (RH)-controlled chamber. The dishes are automatically positioned on the load cell of a microbalance, and the samples are weighed at predefined time intervals (30 min in this study). Samples of approximately 50 mg were used in each measurement, and all mixtures had a molar ratio of 1:1. Isotherms were collected at 25 °C by increasing the RH from 0–95% in 2% steps and a hold time of 10 h at each humidity step. A detailed investigation of the hold time in deliquescence measurements was carried out recently.^25^ The measurement conditions selected in the present study (50 mg sample size and 10 h hold time) are sufficient to detect the deliquescence step in the water uptake curves reliably while still keeping the measurement times acceptable.^25^

### Environmental Scanning Electron Microscopy

Phase transformations on crystal surfaces or at crystal interfaces of salt mixtures were investigated by using environmental scanning electron microscopy coupled with energy-dispersive X-ray spectroscopy (ESEM-EDX, EVO system, Carl Zeiss Microscopy GmbH). Measurements aimed to observe *in situ* topology changes and variations of the elemental composition. To achieve a broad relative humidity range in the high-vacuum chamber, ESEM was performed at 3 °C with the chamber containing only water vapor. Relative humidity was derived from the chamber pressure by RH = (*p*/*p*_sat_) × 100, with *p*_sat_ = 758 Pa at 3 °C. Thus, for example, the working range was 1.3% RH at *p* = 10 Pa and 82.5% RH at *p* = 625 Pa. Pressures were incremented between the minimum and maximum pressures of 10 Pa and 625 Pa, respectively. Each set point was held constant until pressure stability and stage temperature drift of <0.1 °C were achieved before imaging. Other experimental parameters included an accelerating voltage (Extra High Tension, EHT) of 20.00 kV, a LaB6 (lanthanum hexaboride) cathode filament, and an NTS BSD detector (Nano Technology Systems BackScattered). Unless otherwise specified, all micrographs were captured at a magnification of 208×.

ESEM experiments were conducted with a self-grown NaCl single crystal, a NaCl + KCl salt mixture, a mixture of self-grown single crystals of NaCl and KCl and a mixture of NaNO_3_ and KCl. Prior to SEM imaging, the samples were dried at 50 °C and 1% RH for 30 min to remove the adsorbed water accumulated during sample preparation

### Time-Lapse Microscopy

The deliquescence behavior of NaCl, KCl, and their mixture was documented using time-lapse micrographs captured with a 3D digital microscope (HIROX) with the following setting: 100× to 200× magnification, lens MXG-2500REZ; KH-8700, with a view field diameter of 2079.49 and 1.30 μm resolution. The experiments were conducted in a climate-controlled chamber equipped with a glass window, where the relative humidity in a 0.2 L/min constant gas flow of nitrogen was controlled by using a humidity generator GenRH/Mcell (ProUmid GmbH) with an HC2-IC 102 humidity probe (Rotronic). All of the observations were performed at room temperature (20 ± 1 °C).

### Raman Spectroscopy

Raman reference spectra of NaNO_3_ and KNO_3_ were collected on a Senterra Raman dispersive microscope (Bruker Optics GmbH). The laser was operated at 532 nm and 20 mW with an integration time of 10 s, and spectra were recorded in the 20–2500 cm^–1^ spectral range. Additional in situ Raman spectra were collected during the water vapor sorption experiments using a WP785 laser (Wasatch Photonics) operated at 785 nm, 450 mW, and 200 ms integration time (two-scan average). Spectra were recorded in the range 270–2000 cm^–1^ with 7 cm^–1^ resolution and a working distance of 50 mm. The Raman probe was positioned on the glass-top cover of an SPSx-1 μ instrument (ProUmid GmbH, Germany). Spectra were recorded during the water vapor sorption of the NaNO_3_ + KCl mixture, humidity was increased in 2% increments in the range 28–73% RH with a hold time of 5 h at each step.

## Results and Discussion

### Water Adsorption on a NaCl Surface

Micrographs of an NaCl single crystal were collected across a range of relative humidity from 1.3% to 82.5% RH at 3 °C and elemental mapping using energy-dispersive X-ray spectroscopy (EDX) was conducted at each RH step; selected images are shown in Figure 1. Although water was not observed visually on the crystal surface at 1.3% RH, elemental analysis revealed a few oxygen-rich regions, as highlighted by the orange circles in Figure 1c. Given that neither NaCl nor the carbon substrate contains oxygen, these areas are attributed to water molecules either as newly adsorbed water or as residual water after drying. EDX micrographs (Figure 1c–f) demonstrate the variation in oxygen density on the NaCl surface with changing relative humidity. At 75% RH, just below the DRH of NaCl (76% at 3 °C), oxygen (green spots) almost uniformly covers the surface. At 82.5% RH, the crystal surface is dominated by oxygen, indicating the formation of the bulk solution film. Quantitative analysis at 1.3% RH yields weight fractions of 1.6% (oxygen), 54.0% (chlorine), 37.1% (sodium), and 5.6% (carbon substrate). The weight fraction of oxygen increases to 52.8% at 82.5% RH. Figure 1b illustrates the increasing amount of surface water with increasing RH as measured by the oxygen weight fraction, and an EDX elemental mapping image of oxygen is shown in Figure S3. Notably, a change in the slope above 60% RH suggests the beginning of ion mobilization and the formation of a solution film.

**Figure 1:**
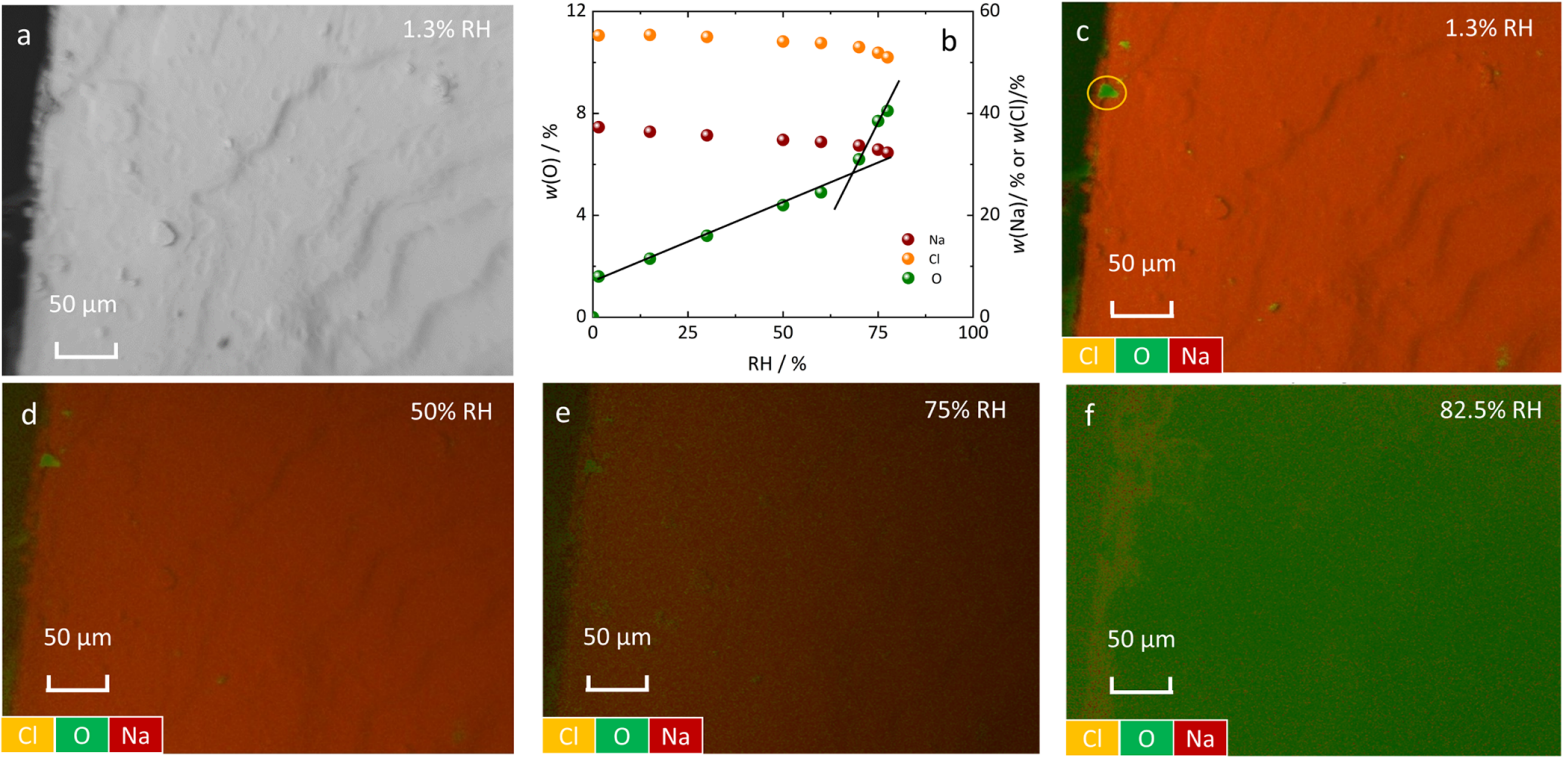
Illustration of the crystal surface topology (ESEM images a) and elemental distribution (EDX images c–f) with changing RH; EDX elemental mapping: sodium (red) chlorine (orange), and oxygen (green); (b) weight fractions of oxygen *w*(O) (H_2_O), sodium *w*(Na) and chlorine *w*(Cl) on the NaCl surface vs relative humidity. Black lines in (b) are drawn to guide the eyes.

Unlike water condensation in the atmosphere or on hydrophobic surfaces, the formation of an adsorbed water film on the NaCl crystal surface helps to bypass the nucleation barrier for water liquefaction. Instead, condensation at higher humidity is driven by the reduced water activity in the brine on the crystal surface rather than by the solid–gas interaction. These results are in line with infrared spectroscopy and X-ray photoelectron spectroscopy studies,^8,11,13^ in which the water adsorption on a NaCl surface has been categorized into three stages: (1) adsorption of isolated water molecules (0–30% RH), (2) multilayer water adsorption (30–50% RH), (3) bulk-like water film formation (50% to DRH). Ion solvation and ion mobility begin below 50% RH, corresponding to approximately two to three water layers on the crystal surface, allowing for the formation of a three-dimensional structure around hydrated ions.^8,11,13^ Enhanced ion mobility facilitates the formation of hydrated ion clusters, increasing the amounts of available sites for further water adsorption, and enhancing water vapor sorption above 60% RH. According to a previous AFM study,^15^ the thickness of this film reaches approximately 5 nm at 75% RH, which is close to the DRH of NaCl at 25 °C. This film thickness is sufficient for ion solvation, thus, it is best considered as a brine film as supported by both experimental observations^11–15^ and thermodynamic considerations.^15^ The investigation of ESEM and EDX elemental mapping offers new insight into the water adsorption behavior on crystal surfaces of ionic solids.

### Mutual Deliquescence of Ternary Salt Mixtures

Water vapor sorption isotherms for pure NaCl, KCl, NaNO_3_, KNO_3_, and their ternary mixtures are shown in Figure 2. It is evident that the mutual deliquescence relative humidity (MDRH) of the four salt mixtures (indicated by the black dots in Figure 2a–d) is lower than the deliquescence humidities (DRH) of the pure salts in all four cases. This shift agrees with both theoretical considerations as discussed below and with the model-calculated MDRH of the four mixtures provided in the Supporting Information (Figures S1 and S2). The depression in MDRH was also confirmed through time-lapse optical microscopy observations of the NaCl + KCl system, as shown in Figure 2e and f. In this setup, adjacent NaCl and KCl crystals simulate a salt mixture, while isolated NaCl and KCl crystals serve as pure salt references for comparison. As illustrated in Figure 2f, the NaCl + KCl mixture begins to transform into a droplet at a certain humidity level, indicating the onset of deliquescence, while the isolated NaCl and KCl crystals remain stable.

**Figure 2:**
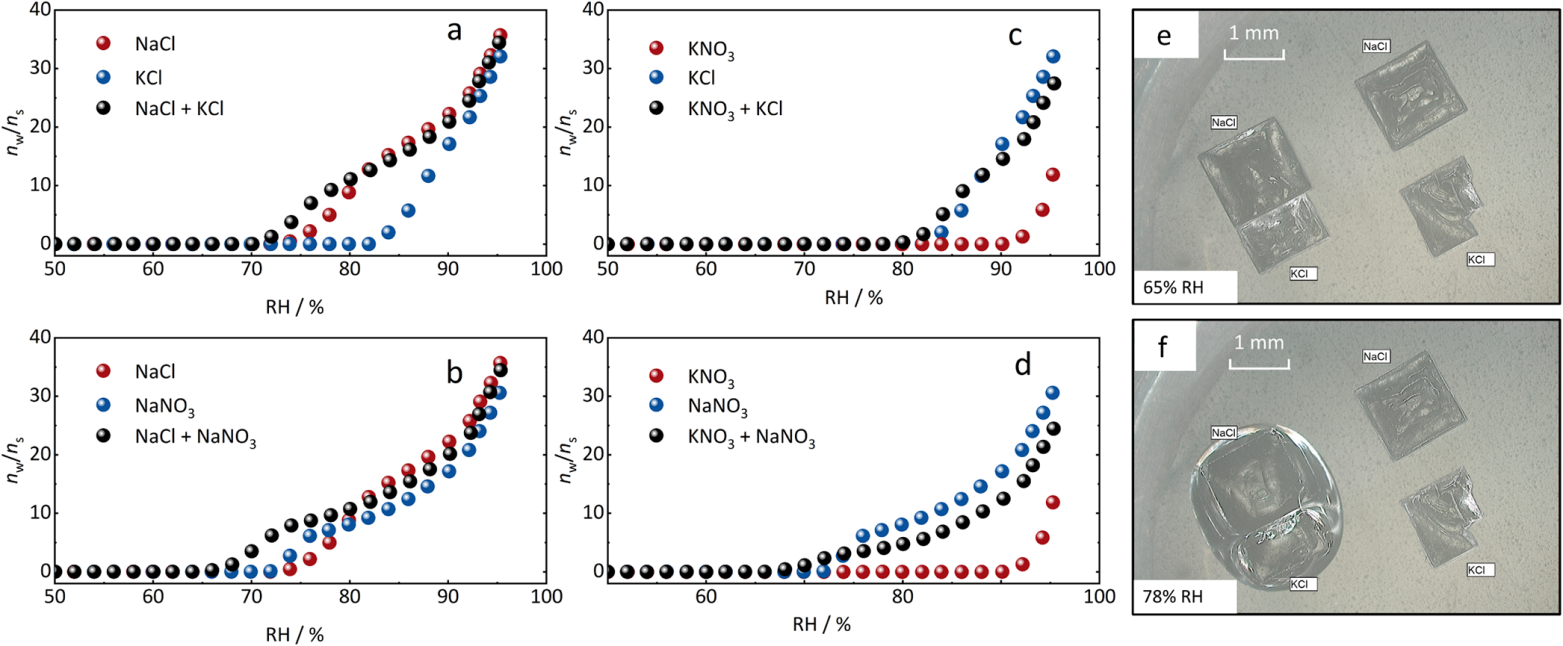
Water vapor sorption curves of pure NaCl, KCl, NaNO_3_, KNO_3_ and their ternary mixtures NaCl + KCl (a), NaCl + NaNO_3_ (b), KNO_3_ + KCl (c), and KNO_3_ + NaNO_3_ (d) at 25 °C. Micrographs show the NaCl + KCl mixture at 65 ± 2% RH (e) and 78 ± 2% RH (f).

To further explore this process on a smaller scale, water adsorption in a NaCl + KCl mixture was observed in ESEM imaging mode at various RHs. Figure 3a shows the initial morphologies of two crystals, KCl (top) and NaCl (bottom), connected by a narrow bridge. The EDX elemental mapping in Figure 3b reveals that certain areas of each crystal were contaminated by the other during sample preparation, visible as red shading on the KCl and the green strip (highlighted by the orange circle) on the NaCl surface. The shape of these contaminated areas, indicated by the variable brightness in the SEM micrograph (Figure 3a, orange circle), aligns well with the EDX image in Figure 3b. Upon exposure to water vapor, the liquid droplet first appears in the contaminated areas at 79% RH, as indicated by the orange circle in Figure 3e. This droplet formation results from the mutual deliquescence between contaminant KCl and the NaCl substrate. However, the droplet remained localized, unable to spread across the entire crystal, as the limited amount of contaminant KCl was quickly depleted, preventing further deliquescence. At the NaCl–KCl interface, the salt bridge begins to become liquid at 80% RH, as highlighted by the green circle in Figure 3f. As time progressed and humidity increased, ions from both KCl and NaCl continuously diffused into the liquid bridge, leading to its expansion and eventually to the liquefaction of the entire system.

**Figure 3:**
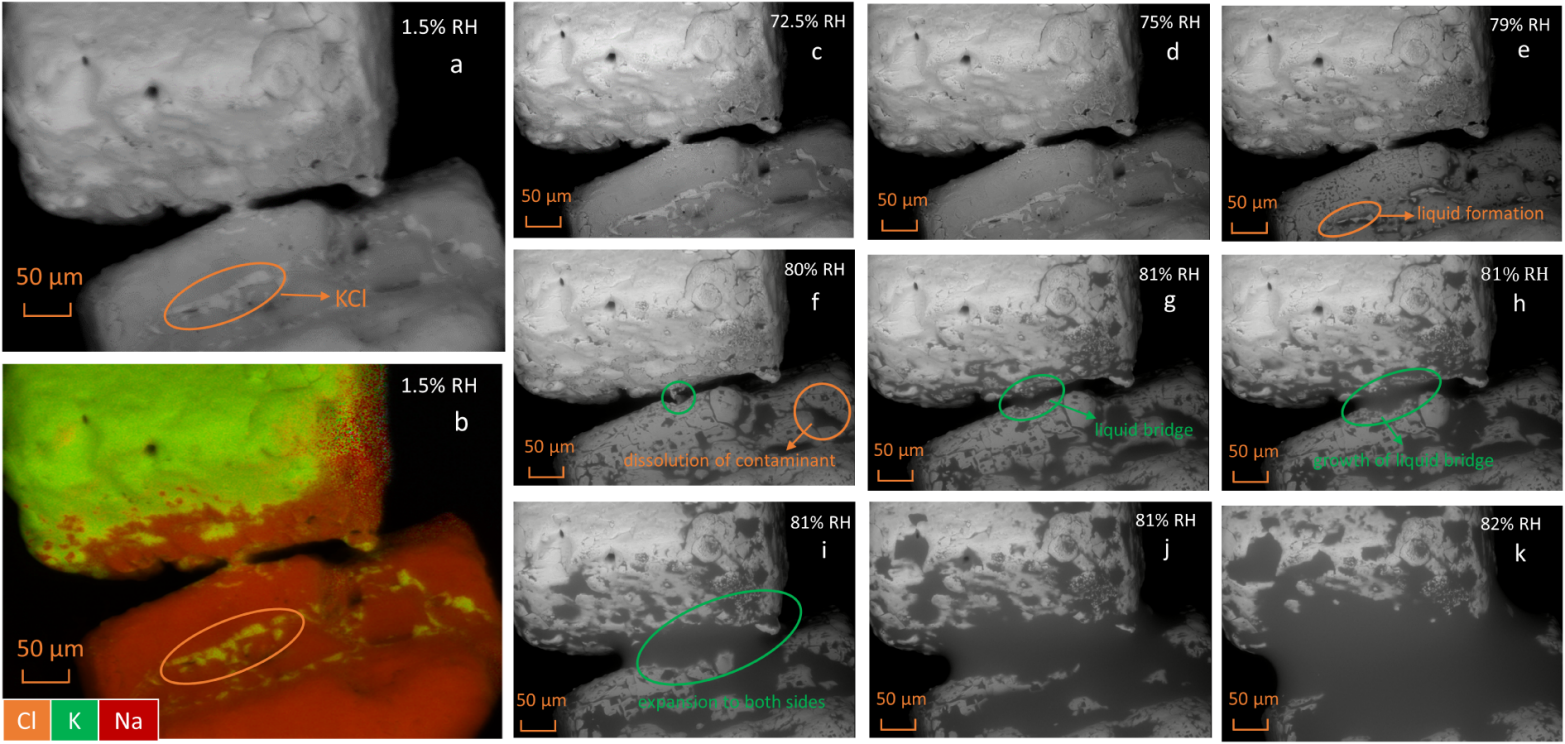
SEM micrograph (a) and EDX elemental mapping (b) of two adjacent NaCl and KCl crystals at 1.5% RH (green: potassium, red: sodium, orange: chloride); (c–k): SEM micrographs of the two crystals at various RHs and 3 °C.

To better assess the process at the interface, a pair of NaCl and KCl crystals with smoother surfaces was grown and placed next to each other (Figure 4). These crystals allow for a contact area closer to that of the standard commercial crystals used before (Figure 3). Water vapor sorption and deliquescence in the interfacial area as observed by SEM are presented in Figure 4 within a humidity range of 75% to 77%. The results indicate that a liquid film forms along the edge of the microcrystal on the crystal surface at 75% RH, as seen in the orange circle in Figure 4a. It is assumed that a liquid film, just as observed in Figure 3 on the crystal surfaces, also forms in the contact area of the crystals, though it is not visible in the micrographs. At 77% RH, the formation of liquid in the interface region is evident and at constant RH the deliquescence process continues.

**Figure 4:**
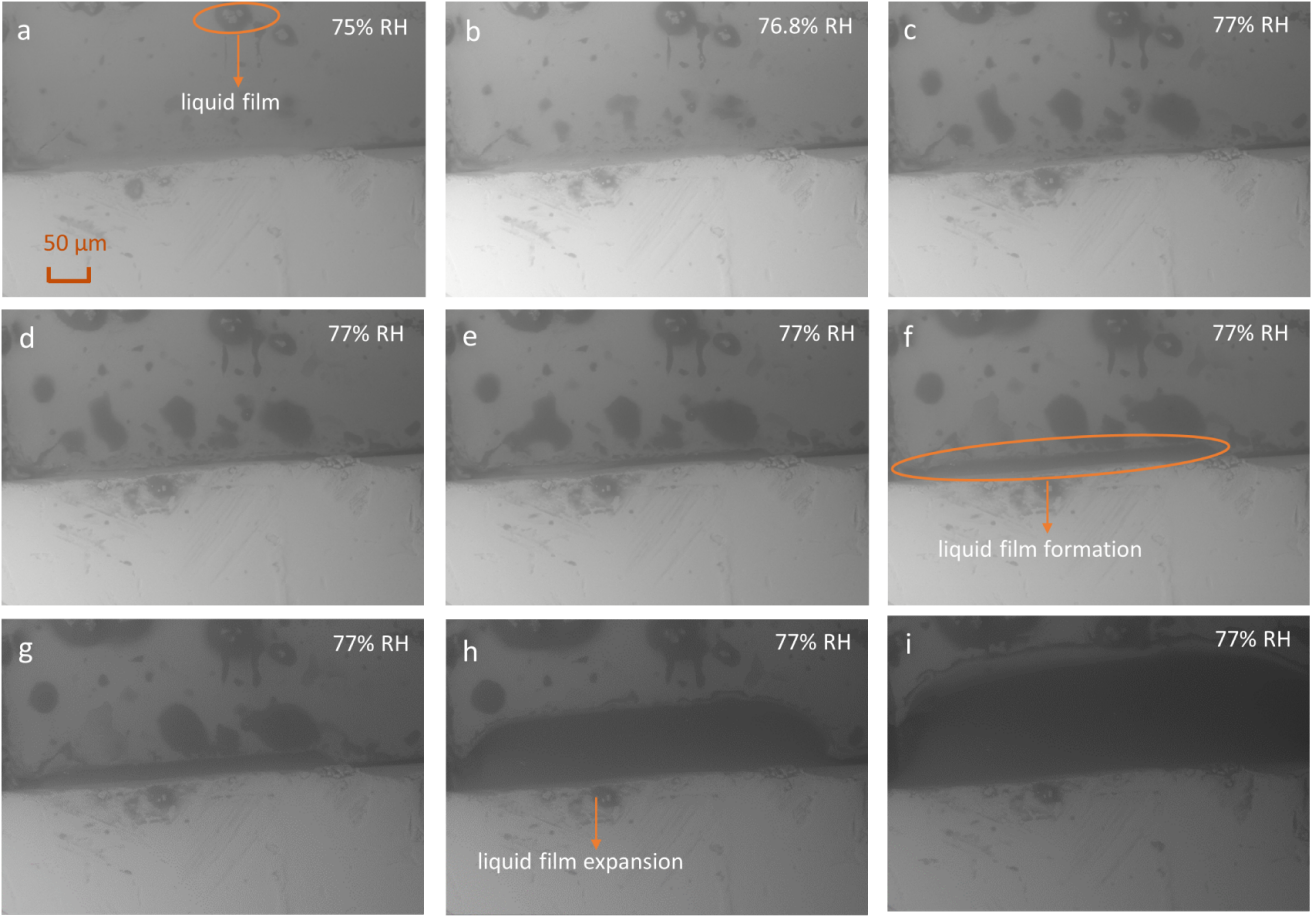
SEM micrographs during the formation of a liquid film at the interface between two crystals of NaCl (top) and KCl (bottom). Micrographs show the interface between the two crystals at 75% RH (a), 76.8% RH (b), and increasing exposure times at 77% RH (c–i); deliquescence starts with the formation of an interfacial liquid film (f) followed by continuous growth of the solution phase (g–i).

To understand why salt mixtures exhibit a lower mutual deliquescence relative humidity compared to that of the respective pure components, it is necessary to interpret this phenomenon from both thermodynamic and dynamic perspectives, focusing on the conditions for equilibrium and the onset of liquefaction at lower humidity. Thermodynamically, deliquescence represents an equilibrium between the crystalline solid, the saturated solution, and water vapor. Thus, the relative humidity equals the water activity of the saturated solution. In this equilibrium, the aqueous solution acts as a bridge that facilitates interaction between water vapor and the salt crystal, maintaining the balance of salt dissolution–crystallization and water evaporation–condensation. Wexler and Seinfeld^26^ demonstrated on thermodynamic grounds, using the Gibbs–Duhem equation, that the water activity of a solution saturated with respect to a solid is always lower in the presence of a second solute. To illustrate this, we consider a saturated aqueous solution of an electrolyte 1 (e.g., NaCl) to which a second electrolyte 2 (e.g., KCl) is added. The Gibbs–Duhem equation for this solution is

1 $$n_{1}d\mu_{1}+n_{2}d\mu_{2}+n_{w}d\mu_{w}=0$$

The chemical potential of NaCl­(aq) does not change and equals the chemical potential of NaCl­(cr) as long as the solution is saturated. At the same time, the chemical potential of KCl­(aq) increases with increasing concentration slowly approaching the chemical potential of the second solid phase KCl­(cr). Thus, the first term in eq 1 vanishes as d*μ*_1_ = 0, the second term is always positive, and, consequently, the third term must be negative. Since $\mu_{w}=\mu_{w}^{◦}+RT\ln a_{w}$ , d*μ*_w_ is only negative if the water activity *a*_w_ decreases upon the addition of the second electrolyte at the saturation of the first one. The same applies if NaCl is added to a saturated solution of KCl. In effect, for both solids, the water activities of saturated solutions decrease with increasing concentration of the second electrolyte. At the intersection of the two curves, both solids are saturated, and the water activity now equals the MDRH (see Figure S2 in the Supporting Information).

Regarding the mechanism of liquefaction at a RH below the MDRH, despite liquid water being thermodynamically unfavorable on a pure salt surface, it is hypothesized that the adsorbed water film acts as a catalyst. As previously explained, the deliquescence of NaCl commences with the formation of a water film. Similarly, water adsorption occurs at the interface of NaCl and KCl. Due to the surface roughness, two crystals are unable to form a perfect contact, likely leaving a slit in the interfacial area, as depicted Figure 5. With increasing humidity, the adsorbed water layers on both sides converge, forming thicker layers, as shown in the ESEM micrographs in Figures 3 and 4. The merged solution film facilitates ion exchange and diffusion between NaCl and KCl. Notably, this merged film contains both Na^+^, K^+^, and Cl^–^ ions, whereas the initial adsorbed water layers on the pure salt surface contain only a single solute. An alternative interpretation suggests that the initial liquid film is formed directly by capillary condensation between two particles.^27^ In porous silica materials, water condensation pressure can drop below 60% RH at 25 °C when the pore diameter is reduced to 2.5 nm,^28^ and this threshold decreases further in the presence of salt.^29^ Although it is unlikely that such a small pore was present in our SEM investigation, nanoscale irregularities likely exist in the contact area of two surfaces, as shown schematically in Figure 5. Thus, the formation of a nanodroplet is essential for connecting two surfaces at the atomic level, regardless of how the droplet was generated. The ion diffusion results in a solution film that is saturated with respect to both salts, exhibiting a lower water vapor pressure than a solution with a single solute. The growth of the solution film is controlled by the humidity and it remains confined to the interfacial area until the RH in the environment reaches the MDRH. At this point, the solution spreads over the bulk crystals, leading to macroscopic dissolution. Microscopic water adsorption or capillary condensation also exists at the grain interface of a pure salt, as shown in the upper schematic diagram of Figure 5. This process acts as a trigger for the transition from water vapor to solution but it remains confined in the interface below the DRH, as the bulk dissolution process is governed by the multiphase thermodynamic equilibrium between gas, liquid, and solid phases.

**Figure 5:**
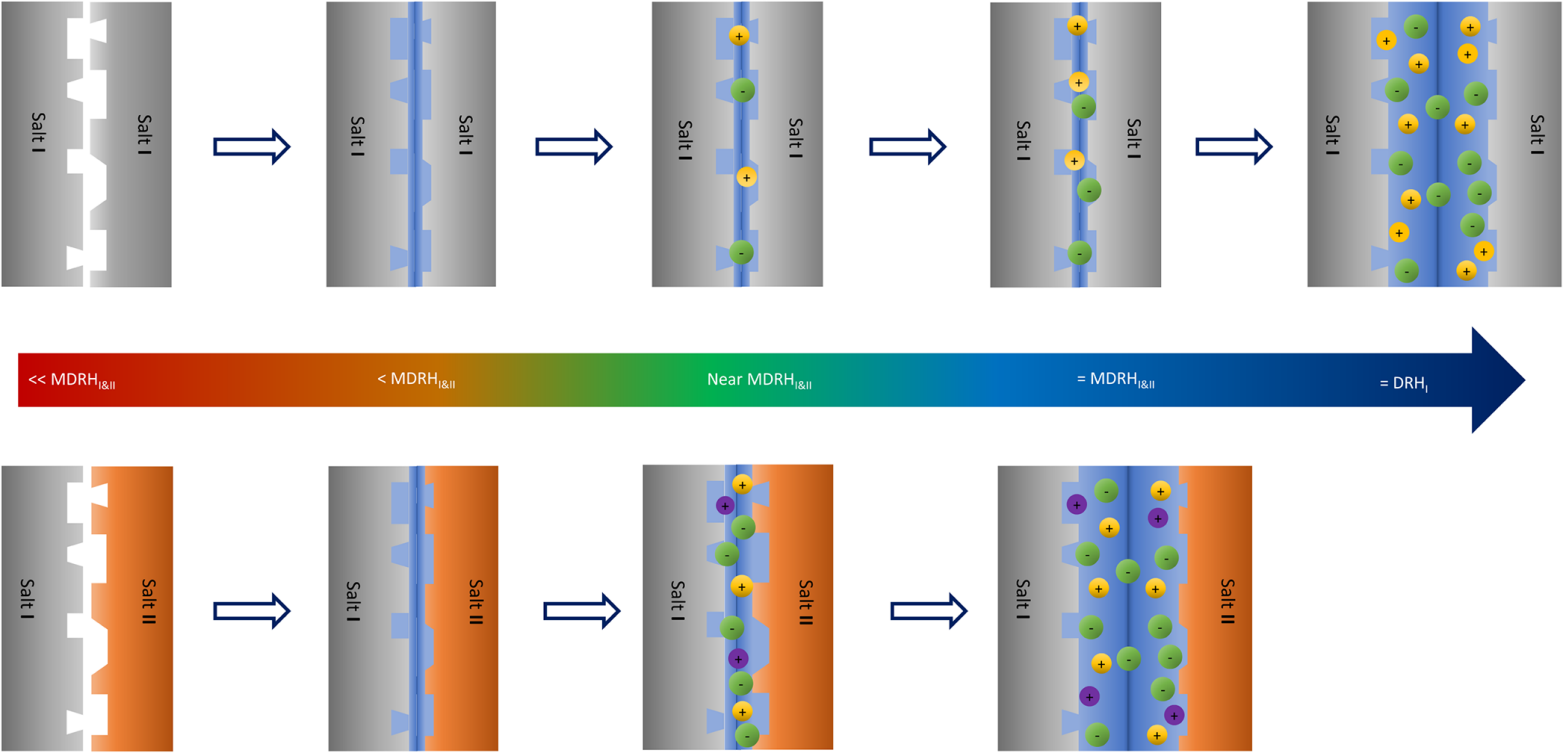
Schematic diagram of the deliquescence of a single salt (top) and a salt mixture (bottom).

### Transformation of a Metastable Salt Pair

The system Na^+^–K^+^–Cl^–^–NO_3_^–^–H_2_O is a simple reciprocal quaternary system. It consists of four solid-phase regions corresponding to NaNO_3_, NaCl, KCl, and KNO_3_, along with five two-phase cosaturated lines and two invariant points (Figure 6). Each pair of salt regions shares a common phase boundary, except NaNO_3_ and KCl, which are separated by NaCl and KNO_3_. Thus, the crystalline phases NaNO_3_ and KCl cannot coexist in equilibrium with a saturated aqueous solution. Instead, they transform to the NaCl + KNO_3_ pair. Consequently, NaCl + KNO_3_ is the thermodynamically stable pair, while NaNO_3_ + KCl is the metastable pair in this reciprocal system. This raises the question of whether this stability preference of the NaCl + KNO_3_ pair is maintained in the absence of liquid water, particularly when the metastable pair NaNO_3_ + KCl is exposed to water vapor.

**Figure 6:**
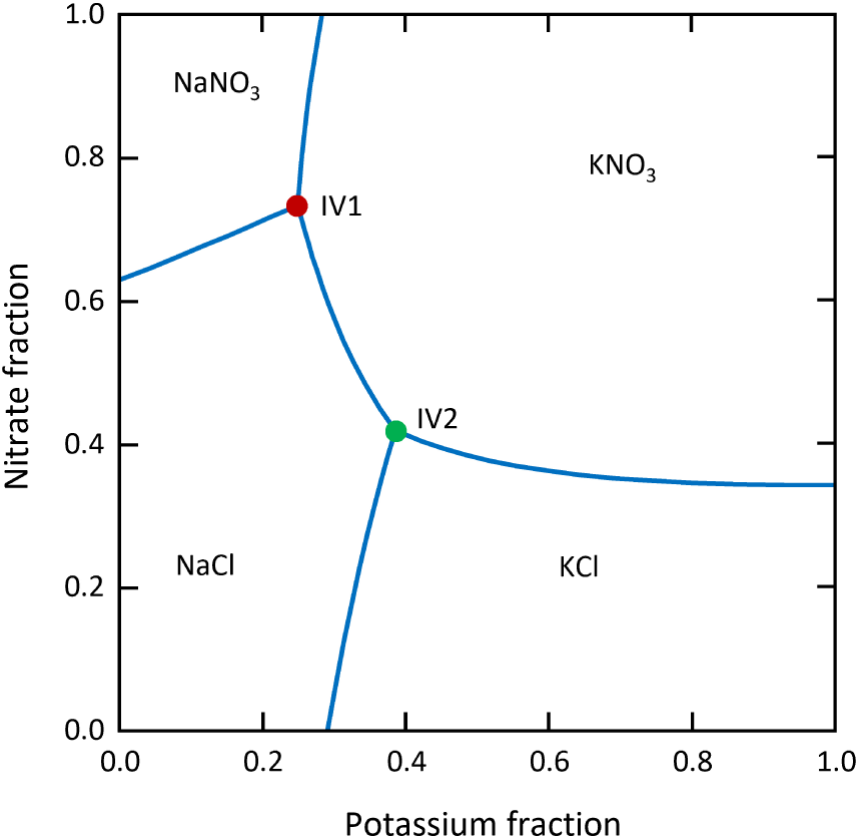
Jänecke diagram of the Na^+^–K^+^–Cl^–^–NO_3_^–^–H_2_O reciprocal system at 25 °C calculated with a thermodynamic equilibrium model based on Pitzer-type ion interaction equations.^30^ Symbols represent invariant points (IV1 and IV2); the model calculated relative humidities in equilibrium with the two invariant points are 62.5% (IV1) and 67.2% RH (IV2), respectively.

The water vapor sorption isotherms of the equimolar mixtures of NaCl + KNO_3_ and NaNO_3_ + KCl, respectively, are depicted in Figure 7a. Notably, the onset of water adsorption for both NaCl + KNO_3_ and NaNO_3_ + KCl mixtures occurs at 68% RH, which is lower than the DRHs of the individual pure components but aligns closely with the humidity of invariant point 2 (NaCl + KNO_3_ + KCl, shown in the phase diagram in Figure 6). Additionally, in a magnification of the results in the humidity range below 68%, a visible water uptake in the sorption curve of NaNO_3_ + KCl is observed (Figure 7b, green dots), initiating at the water activity of invariant point 1 (NaCl + KNO_3_ + NaNO_3_, shown in the phase diagram in Figure 6) at 62% RH. This observation suggests that partial conversion of NaNO_3_ + KCl to NaCl + KNO_3_ occurs at this stage, which is consistent with their common mutual deliquescence relative humidity in the sorption curves. The minor water uptake at 62% is likely due to a transition of the metastable to the stable pair in a dissolution–precipitation process. Similar to the deliquescence of salts and their mixture, also for this metastable salt pair, water adsorption or capillary condensation occurs at the grains at low RH, accompanied by ion solvation within a nanoscale water film or droplet. To be specific, as depicted in Figure 7g, the interfacial solution films originating from both surfaces initially contain only two ions each, either Na^+^ and NO_3_^–^ or K^+^ and Cl^–^. With increasing humidity, the films grow in thickness, merging to form a multicomponent solution containing all four ions supersaturated with NaCl and KNO_3_. As a result, NaCl and KNO_3_ crystallize from the solution film, until KCl is depleted. Prior to reaching a new equilibrium, all four solid phases, NaCl, KNO_3_, NaNO_3_, and KCl temporarily coexist within the interfacial region. This phenomenon may explain the minor water uptake at 62% RH in the sorption curve of Figure 7b, which reflects the mutual deliquescence of a NaCl, NaNO_3_, and KNO_3_ mixture. The extent of mutual deliquescence is determined by the relative rates of precipitation (of NaCl and KNO_3_) and dissolution (of NaNO_3_ and KCl). In the 62–68% RH range, the bulk solution, rather than the microscopic solution film, serves as the reaction medium, facilitating interaction between the two crystals.

**Figure 7:**
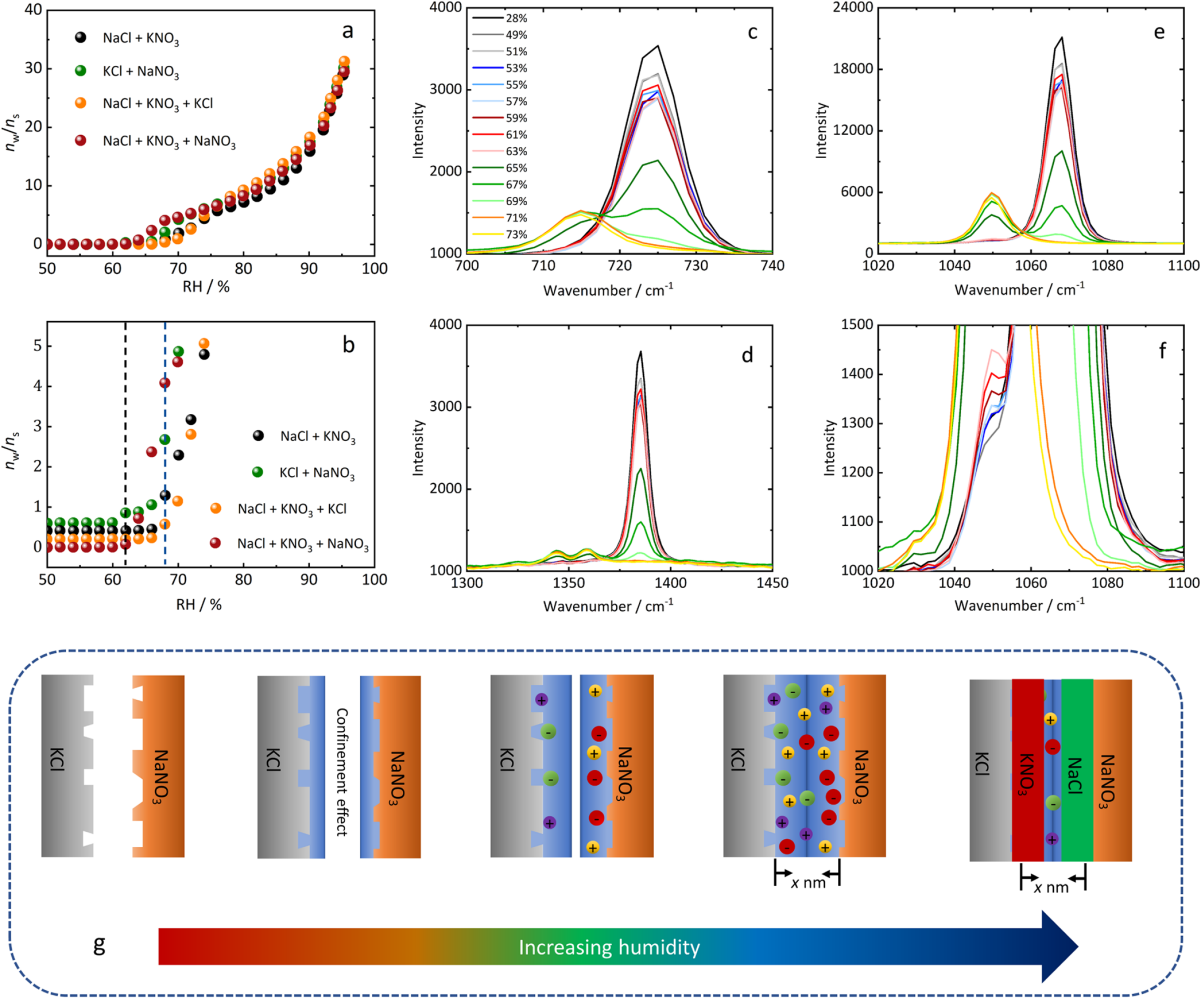
(a): Water vapor sorption curves of several binary and ternary salt mixtures of the system Na^+^–Cl^–^–K^+^–NO_3_^–^–H_2_O; (b): magnification of the RH range in which deliquescence occurs (data are displaced vertically for clarity by 0.2 (orange), 0.4 (black), and 0.6 (green); (c–e): Raman spectra of the NaNO_3_ + KCl mixture at various RH; (f): magnification of the spectra in the range of 1020–1100 cm^–1^; (g): schematic diagram of the transition from the metastable to the stable pair.

To validate the proposed hypothesis, Raman spectra of the metastable NaNO_3_ + KCl pair, with a molar ratio of 1:1, were recorded over a humidity range from 28% RH to 73% RH at 25 °C. Three spectral regions, specifically 700–740 cm^–1^, 1020–1100 cm^–1^, and 1300–1450 cm^–1^, are illustrated in Figure 7c, d, e, and a detailed view of the Raman peak at 1050 cm^–1^ is presented in Figure 7f. All observed Raman spectra are attributed to the fundamental vibrational bands of NO_3_^–^ and exhibit notable changes above 63% RH. Indeed, a subtle shoulder appears at 1050 cm^–1^, whose intensity slightly increases with increasing humidity until a visible peak at approximately 65% can be observed. By comparing them with the reference spectra of NaNO_3_ and KNO_3_ in Figure 8, we deduce that this peak shift reflects the transformation of NaNO_3_ into KNO_3_. Although NaCl is not detectable in the Raman spectrum, it is highly probable that it precipitates as well to maintain liquid–solid equilibrium. The onset of these obvious changes in the Raman spectra falls between the MDRH of invariant point 2 and the invariant point 1 (shown in the phase diagram in Figure 6), in accordance with the narrow humidity window for water uptake by the NaNO_3_ + KCl system. This supports our assumption that adsorbed water provides the necessary liquid environment for the reaction NaNO_3_ + KCl → NaCl + KNO_3_. This transformation can be divided into two stages: the formation of a nanoscale film and the development of a bulk solution. The initial shoulder in Figure 7f corresponds to the formation of a small amount of KNO_3_ within a thin solution film, which results in a minor increase in water uptake at 62%, as seen in the water vapor sorption curve in Figure 7a. This limited water uptake occurs because mutual deliquescence is impeded once one of the eutonic components is depleted. In the second stage, the presence of a bulk solution, following mutual deliquescence, the dissolution–precipitation reaction is significantly accelerated compared to the nanoscale film stage, leading to the pronounced Raman peak at 1050 cm^–1^, characteristic of KNO_3_. Additional Raman measurements using a Raman microscope with higher resolution and confirming the results presented in Figure 7 are provided in the Supporting Information (Figure S4).

**Figure 8:**
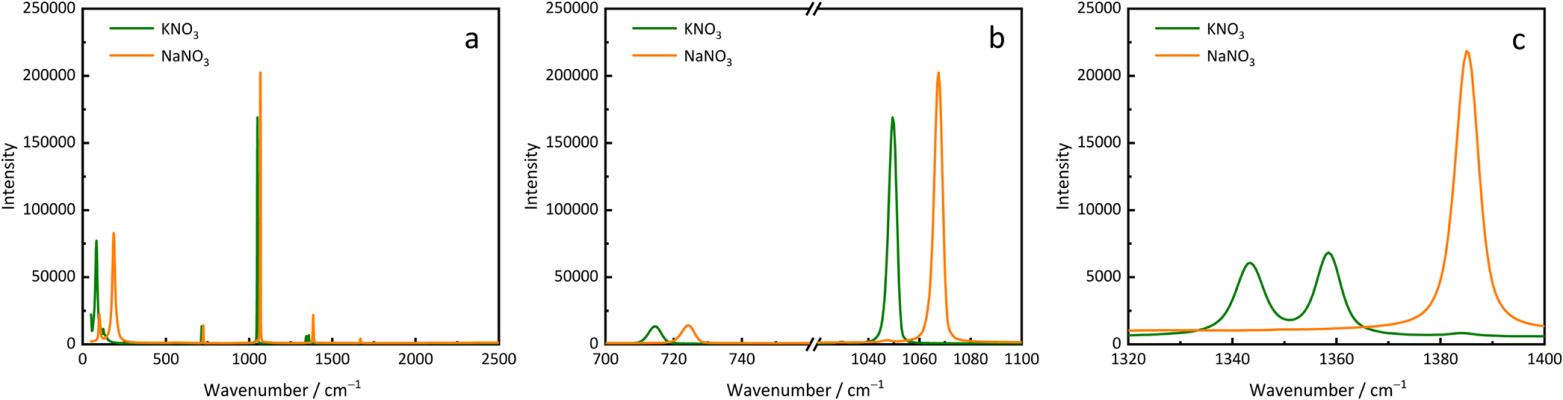
Raman reference spectra of pure NaNO_3_ and KNO_3_ (collected with the Senterra Raman microscope); (a) full-range spectrum, (b) ranges 700–760 cm^–1^ and 1020–1100 cm^–1^, (c) range 1320–1400 cm^–1^.

The ESEM images in Figure 9a–d illustrate the morphological changes occurring as water molecules adsorb onto the salt surface. It is worth mentioning that two images were captured at 62% RH within a 12 h interval, with the latter labeled as 62% RH-2 (Figure 9c). It is clear from Figure 9a that the NaNO_3_ surface appears generally smooth at 5% RH, although some irregularities are visible. When the humidity reaches 62%, minimal visible changes are observed initially. However, after 12 h, the flat surface becomes covered with microcrystals, which is likely to be KNO_3_, and a liquid film is seen at the interface of the two crystals (Figure 9c). According to the sodium and potassium distributions from EDX mapping (Figure 9e), the KCl crystal surface is coated with sodium in several small spots. These sodium-rich spots expand into a larger area at 62% RH, marked by red zones in Figure 9f. The comprehensive elemental mapping image in Figure 9h confirms that the red areas represent NaCl microcrystals, as they contain sodium and differ visibly from NaNO_3_. The greenish surroundings of the NaCl microcrystals originate from water-related oxygen, indicating the formation of a solution film. Additionally, the dark gray regions near the interface of the two crystals, such as the area circled in orange in Figure 9c, further suggest the presence of a solution film. This observation aligns with the small increase in water uptake at 62% RH shown in Figure 7b. Moreover, the Raman spectra presented in Figure 7 indicate the appearance of KNO_3_, the second reaction product next to NaCl, which corresponds to microcrystal formation on the NaNO_3_ surface. Over time and with increased humidity, the entire surface becomes coated with newly generated KNO_3_ at 74% RH.

**Figure 9:**
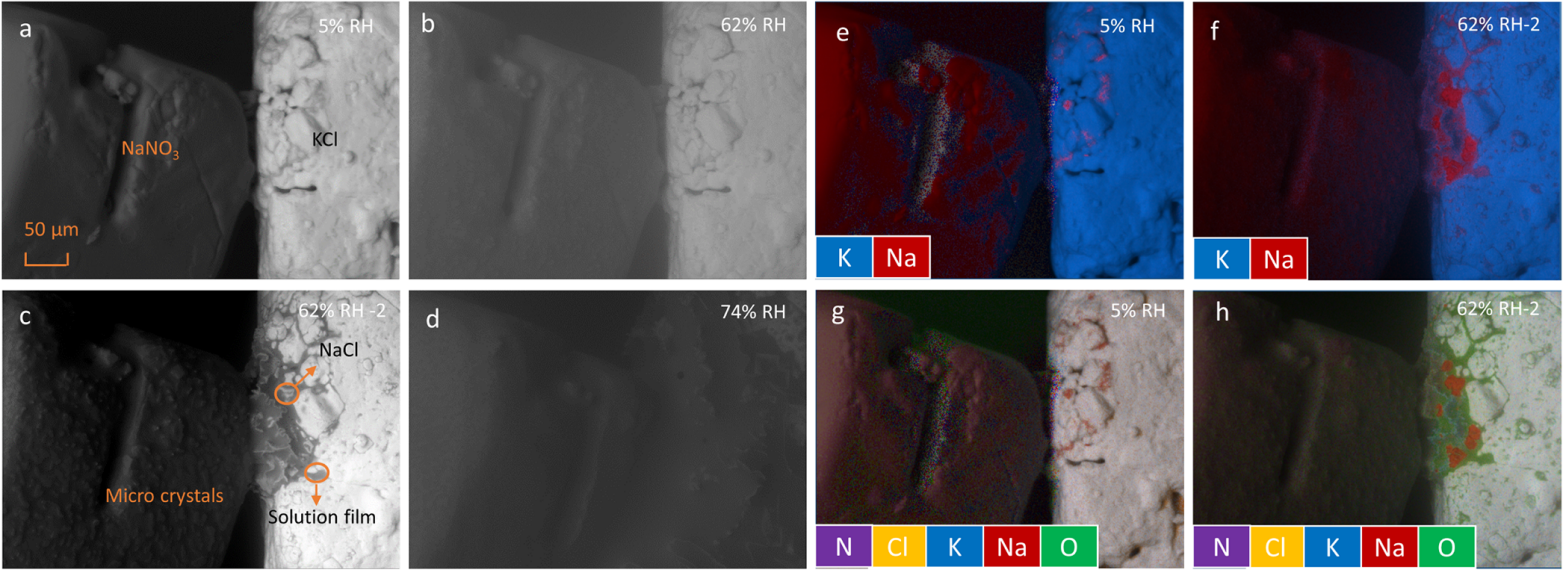
ESEM and EDX images of the NaNO_3_ + KCl mixture at different RH. (a–d): morphology of the salt crystals; (e–h): elemental distributions, red: sodium, purple: nitrogen, orange: chloride, blue: potassium, green: oxygen, (e–f): without oxygen, (g–h): with oxygen.

## Conclusions

In this study, the interactions between water vapor and salts or salt mixtures were investigated using water vapor sorption, optical microscopy, environmental SEM, and Raman spectroscopy. The quaternary reciprocal system Na^+^–Cl^–^–K^+^–NO_3_^–^–H_2_O and its respective subsystems were selected for this investigation. Using ESEM and EDX elemental mapping, the formation and evolution of a water film on a NaCl surface were confirmed in response to increased humidity as observed previously using other experimental techniques.^8–15^ Subsequently, the shift of the mutual deliquescence humidity of salt mixtures to below the DRH of the pure salts was observed by using dynamic water vapor sorption. This shift is in agreement with previous experimental results and with theoretical considerations;^5,23,26^ it is also validated by model calculations. Using ESEM and optical microscopy, the findings indicate that it is the direct contact of adsorbed water films on two adjacent crystal surfaces or the capillary condensation in void spaces at grain interfaces that leads to a mixed electrolyte solution film with lower water activity serving as the initiator for mutual deliquescence.

The transition from the metastable salt pair NaNO_3_ and KCl to the thermodynamically stable pair NaCl and KNO_3_ in the quaternary reciprocal system occurs in two steps upon exposure to water vapor: (1) dissolution of NaNO_3_ and KCl in the interfacial water film; (2) the precipitation of NaCl and KNO_3_. It is important to note that this transition provides an example of a solid-state reaction that is significantly accelerated by the formation of a nanoscale water film, although water is not involved in the reaction equation. Overall, the water film plays a crucial role at solid–water vapor interfaces and as an ion diffusion medium. Given the ubiquitous presence of water vapor in natural and industrial environments, it is likely that such a water film forms on the surface of most materials, thus triggering deliquescence, caking, or chemical reactions at solid–solid interfaces. Such processes may have significance in many disciplines including geochemistry, atmospheric chemistry, astrobiology, pharmaceutical and food technology, or other industrial applications. The observed phenomena underscore the essential role of water films in phase transitions of salt mixtures, and these universally existing interactions between water vapor and soluble electrolytes warrant further exploration.

## Supplementary Material



## Notes

Model calculations (solubilities of the ternary systems investigated with corresponding water activities of the saturated solutions) and additional experimental results (oxygen mapping on NaCl crystals at different relative humidities, Raman spectra recorded during the transformation of the metastable salt pair NaNO_3_ + KCl) (PDF)
