# Supplementary material for: Water Films: The Motor of Phase Transitions in Salt Mixtures
Source: Langmuir. 2025 Nov 13;41(46):30938–47. doi: 10.1021/acs.langmuir.5c02607 (PMC12659436; doi:10.1021/acs.langmuir.5c02607)
Supplement: Supplementary file 1 [file la5c02607_si_001.pdf]

Supporting information

Water Films: The Motor of Phase Transitions in Salt Mixtures

Shaoheng Wang[1], Sebastiaan Godts[2], Amelie Stahlbuhk[1], Michael Steiger[1]*

[1]Department of Chemistry, University of Hamburg, Germany

[2]Royal Institute for Culture Heritage (KIK-IRRA), Belgium

* Correspondence tor: Michael.Steiger@uni-hamburg.de

Number of pages: 4
Number of figures: 4

Contents

1 Phase diagrams of ternary systems 1

2 Additional experimental data 3

2.1 Oxygen mapping on NaCl surfaces

2.2 Raman microscopy measurements

1. Phase diagrams of ternary systems

The solubility diagrams of the ternary systems $NaCl–KCl–H_2O$, $NaCl–NaNO_3$, $NaNO_3–KNO_3–H_2O$, $KCl–KNO_3–H_2O$ have been calculated using the same thermodynamic model[1] used to calculate the phase diagram of the reciprocal system (Figure 6 in the main article). This model has been validated using experimental solubility which are reproduced to within experimental data by the model calculations.[1] The solubilities in the four ternary systems are presented in Figure S1. Each line represents the solubility of one salt upon increasing the concentration of the second salt. The intersection of the two curves represent the invariant points (IV), i.e., the solutions saturated with respect to both salts. Figure S2 presents the water activities of the saturated solutions shown in Figure S1 versus the mole fraction composition, i.e., the relative humidity in equilibrium with these solutions. Again, the intersection of two respective curves is the equilibrium humidity (water activity) of the solutions that are saturated with respect to both solids. At lower relative humidities, a solution is not stable and both salts are in the crystalline state. Therefore, the water activity of the solution saturated with both salts equals the mutual deliquescence humidity (MDRH) of the respective mixture, i.e., the relative humidity at which deliquescence starts. In agreement with theory, it is obvious the saturation water activities decrease with increasing concentration of the second salt such that the MDRH is always lower than the DRH of both single salts. The calculated values of the MDRH are in good agreement with the experimental data shown in Figure 2 of the main article, in particular, if it is considered that the water vapor sorption measurements are dynamic rather than equilibrium measurements.

1 Steiger, M.; Kiekbusch, J.; Nicolai, A. An improved model incorporating Pitzer's equations for calculation of thermodynamic properties of pore solutions implemented into an efficient program code. *Constr. Build. Mater.* **2008**, *22*, 1841–1850.

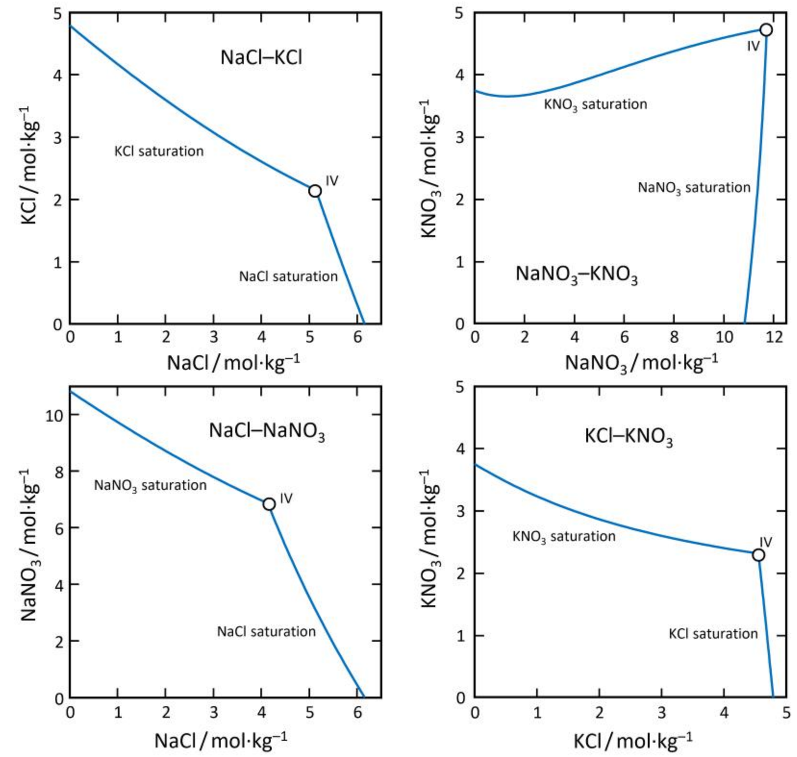

Figure S1. Solubilities in the four ternary systems, solid phases as indicated (symbols represent invariant points, IV).

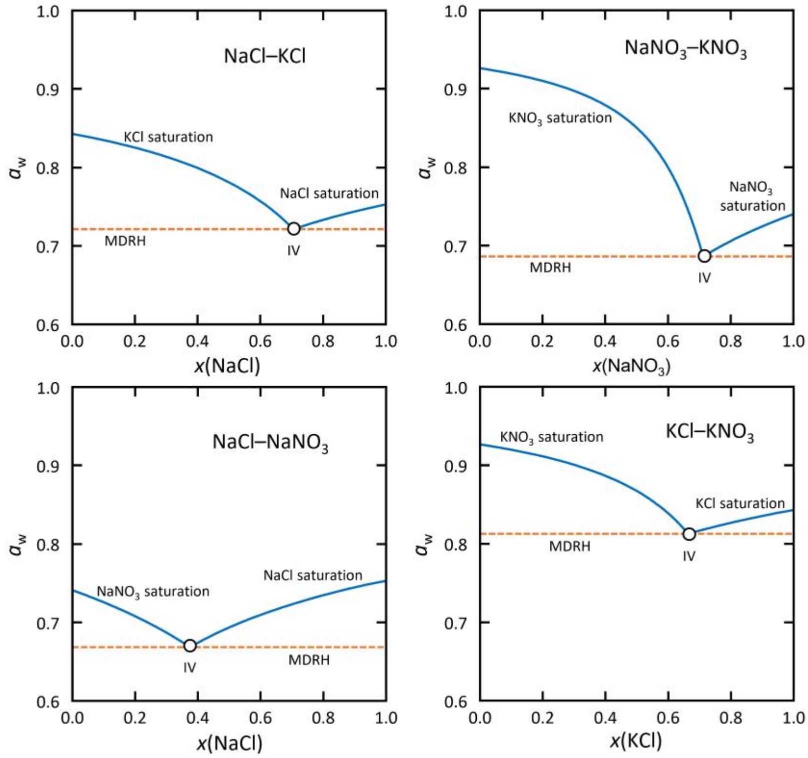

Figure S2. Water activities a_w of saturated and mutual deliquescence humidities (MDRH), a_w at the invariant points.

2. Additional experimental data

2.1 EDX images of oxygen distribution on the NaCl surface

Figure S3 shows the results of the elemental mapping of oxygen on the NaCl surface using energy-dispersive X-ray spectroscopy (EDX) conducted during the ESEM experiment at various relative humdities (Figure 2 of the main article). With increasing relative humidity, there is continuous increase in the amount of oxygen, representing water molecules, on the crystal surface. Quantitative analysis (Figure 1b in the main article) confirm that there is a more pronounced increase above 60% RH indicating beginning ion mobilization and formation of a solution film.

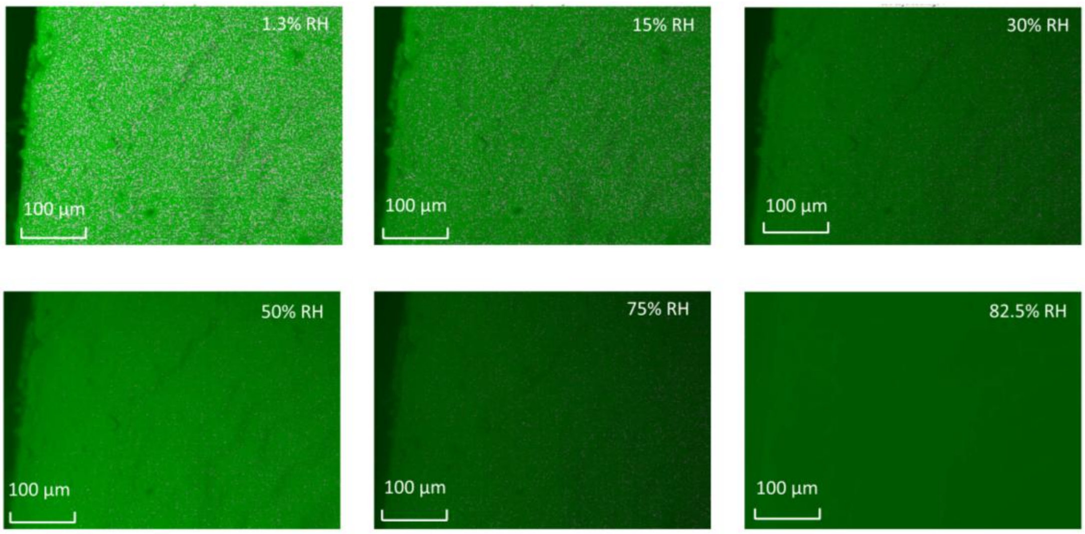

Figure S3. EDX elemental mapping of oxygen distribution on the NaCl surface as the relative humidity increases from 1.3% to 82.5 % RH.

2.2. Raman microscopy measurements

Additional Raman spectra were collected on a Senterra Raman dispersive microscope (Bruker Optics GmbH). The laser was operated at 532 nm and 100–400 mW with an integration time of 10 s and spectra were recorded in the 200–2000 cm^{-1} spectral range. The Raman microscope was equipped with a THMS600 stage (Linkam Scientific Instruments) for temperature and humidity control. An air flow with controlled RH at 25 °C was provided by a humidity generator MHG32 (ProUmid GmbH). During collection of the Raman spectra, the laser was focused on KCl cyrstals in contact with $NaNO_3$ crystals. Spectra were recorded at 50% and 70% RH, respectively. By comparing the micrographs Figure S4a (50% RH) and Figure S4b (70% RH), it is evident that new microcrystals have formed on the KCl surface upon increasing the RH to 70%. This is confirmed by the corresponding Raman spectra shown in Figures S4c–d, where characteristic Raman signals of crystalline KNO_3 only appear in the spectrum recorded at 70% RH (shown in black). Thus, these measurements confirm the in situ Raman measurements shown in Figure 7 of the main article.

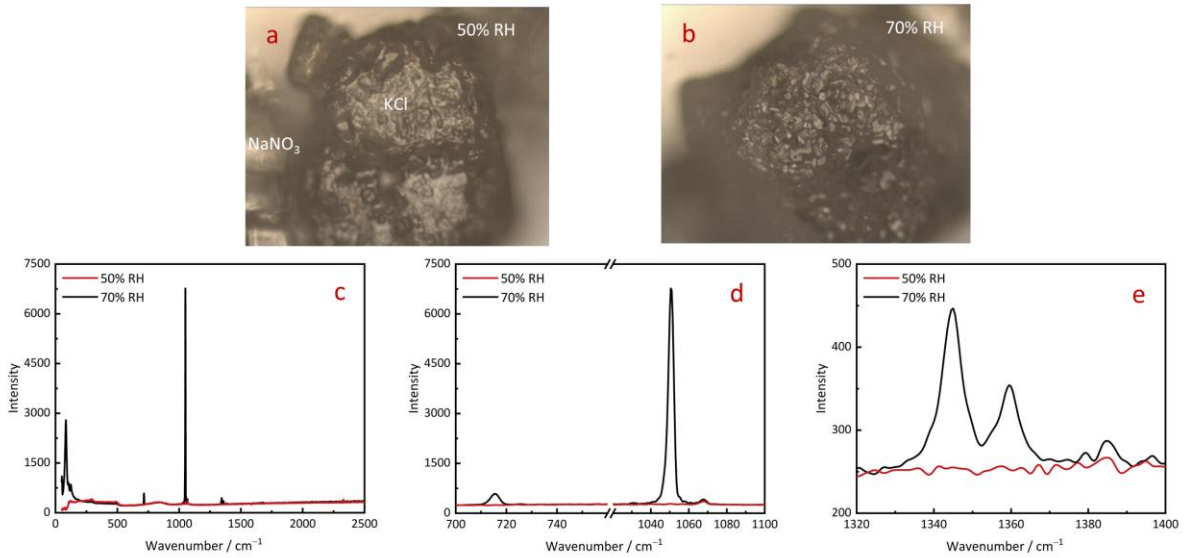

Figure S4. Raman spectra recorded during exposure of an initial mixture of pure $NaNO_3$ + KCl to 50% and 70% relative humidity, respectively. (a)–(b): Optical micrographs of KCl crystals during Raman measurements at the corresponding relative humidities; (c)–(e): Raman spectra recorded at 50 % RH (red) and 70% RH (black) confirming the formation of KNO_3 microcrystals on the KCl crystal surface in contact with $NaNO_3$ at 70 % RH.
